# Predictors of institutional delivery service utilization among women of reproductive age in Gambia: a cross-sectional analysis

**DOI:** 10.1186/s12884-020-02881-4

**Published:** 2020-03-30

**Authors:** Sanni Yaya, Ghose Bishwajit

**Affiliations:** 1 School of International Development and Global Studies, University of Ottawa, Ottawa, Canada; 2 The George Institute for Global Health, The University of Oxford, Oxford, UK

**Keywords:** Health facility delivery, C-section, Maternal health, Gambia, global health, Health service utilization, women’s health

## Abstract

**Background:**

Over the last two decades, Gambia has made noticeable progress in the reducing the high maternal mortality rates and improving child survival rates. Nonetheless, numerous infrastructural and financial constraints continue to restrict access to institutional delivery care, a key component of achieving the maternal and child health related Sustainable Development Goals (SDG 3.1). This study assesses factors that predict women’s choice of mode and place of delivery in urban and rural Gambia.

**Methods:**

Cross-sectional data from the latest round of Gambia Demographic and Health Survey (2013) on women aged 15–49 years (*n* = 5351) were analyzed. The outcome measures were place (home vs health facility) and mode of delivery (caesarean vs normal) in urban and rural Gambia. Data were analyzed using descriptive and multivariate regression methods.

**Results:**

About three-fifth (60.8%) of the participants had their last childbirth at a health facility and 39.2% at their home. There was a significant urban-rural difference in the prevalence of facility delivery with 86.9% of the urban women choosing health facility over home compared with 45.8% among the rural women. In the regression analysis, place of residence, education of participants and the husband, employment, parity and use of antenatal care were significantly associated with the use of health facility delivery services. For instance, having secondary [OR = 1.657, 95%CI = 1.337,2.053] and higher education [OR-2.451, 95%CI = 1.166,5.150] showed higher odds for using facility delivery services; and women from the richest wealth quintile had significantly higher [OR = 2.239, 95%CI = 1.525,3.289] odds of using facility delivery compared with those in the lowest quintile.

**Conclusion:**

Our findings suggest a sub-optimal use of professional childbirth services among Gambian women which appears to be driven by various geographical, educational, wealth inequality, parity and low use of ANC services. Addressing the socioeconomic and demographic inequalities may lead to a more widespread usage of maternity services in Gambia.

## Introduction

Gambia, a former British colony, is the smallest country on mainland Africa and is one of the most densely populated countries in West African region. The population is predominantly rural and is dependent mostly on subsistence agriculture. The demography of the country is characterized by a relatively young age structure, low life expectancy, widespread malnutrition, higher maternal and child mortality (MCM) rates [1–4]. Driven by the internationally set initiatives and targets, the country has been able made significant strides in its attempt to reduce the burden of MCM, however was unable to meet the maternal and child health related Millennium Development Goals (https://bit.ly/2I1p5GZ) [5]. Maternal mortality stands at 706 per 100,000 live births (in contrast with the MDG target of 75) and infant mortality rate at 69 per 1000 live births (in contrast with the MDG target of 67) [6]. The healthcare system is struggling to meet the most basic needs such as improving the coverage of family planning and reproductive healthcare services [5, 7]. Regardless of the success in the last two decades, the country still lags behind in its commitment to make the necessary intervention strategies to tackle the high maternal mortality rates.

The risk of complications and fatal outcomes are multidimensional and are associated with various demand (such as low awareness of the risk factors and care-seeking behaviour) and supply-side factors (such as inadequate healthcare facilities, poor quality of services). Addressing the barriers to using skilled birth assistance (SBA) is central to controlling the burden of MCM in developing countries. A large body of research evidence exits on the beneficial role of using SBAs in averting preventable maternal deaths. A less commonly studied topic in the area of maternal healthcare in Africa is the use of C-sections which is more common in the middle- and high-income countries. In certain cases, especially in the complicated scenarios where regular vaginal delivery is considered to be threatening for the survival of the mother and the child (e.g. obstructed labor and other emergency obstetrical conditions), C-sections provide a safer but pricier alternative [8]. As per the recommendation of the World Health Organization (WHO), the prevalence of C-section should remain within the range of 10–15% of the total deliveries, and should be carried out only under specific medical conditions [9]. Although C-section is not without its own disadvantages, such as high cost and complications arising from the incision, an increasingly larger proportion of women are choosing C-section over normal delivery for several reasons such as avoiding injuries in the birth canal and other medical reasons. However, C-section is a far more expensive process and can incur significant financial burden for the mother and her family especially in low-income settings like Gambia where health insurance coverage is very low. Women lacking the financial resources to undergo C-section might end up choosing the normal or unassisted birth even when a C-section would seem mandatory.

Previous studies have attempted to explore the determinants of using professional childbirth services from various perspectives: behavioral, cultural, economic, and sociodemographic factors at individual level and remoteness of health facility, inadequate infrastructure and skilled human resource for healthcare at community level [10–14]. Although a large number of studies have been conducted to explore the reasons behind the under- and non-use of SBAs in countries from Africa and other developing regions, not much is known about the use of professional childbirth services Gambia, especially the use of C-section. Understanding the sociodemographic inequalities can facilitate devising policies and actions to support the usage of professional childbirth services in the country. Therefore, the present study was conducted to address this research gap using nationally-representative data from Gambia Demographic and Health Survey (DHS) conducted in 2013. This was a cross-sectional survey that provided information on a wide range of demographic and socioeconomic variables. These variables were selected systematically to fit within the scope of the Andersen and Newman Behavioural Model. These findings will help advance the understanding of the sociodemographic inequalities in the uptake of SBAs and C-section services in Gambia as well as in the neighbouring countries with similar economic and sociocultural environment.

## Methods

### Data source

Data for this study were collected from Gambia Demographic and health survey (GDHS 2013). The survey was implemented by Gambia Bureau of Statistics and the Ministry of Health and Social Welfare. Technical assistance came from ICF International and financial support from the government of The Gambia, the U.S. Agency for International Development (USAID), the United Nations Population Fund (UNFPA), the United Nations Development Programme (UNDP), the United Nations Children’s Fund (UNICEF), the Joint United Nations Programme on HIV/AIDS (UNAIDS), the World Health Organization (WHO), and the Global Fund. provided through the worldwide Demographic and Health Surveys programme. The main purpose of DHS surveys is to provide quality information for monitoring and evaluation of population health programmes and assist in evidence-based health policy making. For this survey, sample population were selected from 14 sampling stratum divided into 281 Enumeration Areas or clusters (also known as primary sampling units) throughout the eight regions (known as Local Government Areas).

DHS surveys use multistage sampling strategy for sample selection. In the first stage, the Enumeration Areas are selected with probability proportional to size and with independent selection in each sampling stratum. After selection of the Enumeration Areas, 25 households per Enumeration Area using equal probability systematic selection. A total of 105 interviewers and supervisors were recruited for training and the training of was conducted from November 26 to December 14 of 2012. Data collection for the survey took place from February 2 to April 28 of 2013. 10,233 women were interviewed with a response rate of 90.7%. Further details of the surveys are available from the final report: The Gambia Bureau of Statistics (GBOS) and ICF International. 2014. The Gambia Demographic and Health Survey 2013. Banjul, The Gambia, and Rockville, Maryland, USA: GBOS and ICF International.

### Outcome measures

The outcome variables of interest were: 1) place of delivery: home vs health facility, 2) use of C-section: yes vs no.

### Explanatory variables

Selection was explanatory variables was guided by Andersen’s behavioral model of health service utilization which postulates that healthcare utilization is a function of three major factors: 1) predisposing factors, 2) enabling factors and 3) need factors [15]. For this study, the data were secondary and hence the selection of the explanatory variables in line with the behavioral model was not possible. Based on the availability in the dataset, the following are included in the analysis: Age (15–19, 20–24, 25–29, 30–34, 35–39, 40–44, 45–49); Residency (Urban, Rural); Education (No Education, Primary, Secondary, Higher); Husbands education (No Education, Incomplete Primary, Incomplete Secondary, Higher); Employment (Not Working, Professional/Technical/Managerial, Agricultural - Self Employed); Wealth quintile (Poorest, Poorer, Middle, Richer, Richest); Access to electronic media (No, Yes); Heard of FP on internet (No, Yes); Religion (Islam, Other); Ethnicity Mandinka/Jahanka, Wollof Jola/Karoninka, Fula/Tukulur/Lorobo, Serahuleh Other); Parity (1–5, > 5); Household head (Male, Female); Child wanted (Wanted Then, Wanted No More).

### Data analysis

Data were analyzed with Stata version 14. Dataset was cleaned by applying the inclusion criteria: experience of at least 1 childbirth in the preceding 5 years. As the surveys used cluster sampling techniques, all analyses were adjusted for this by using the *svy* command [16]. This command uses the information on sampling weight, strata, and primary sampling unit provided with the datasets. Sample characteristics were described as frequencies and percentages. Prevalence of using facility delivery and C-section (for total, urban and rural sample) was presented as bar charts. The predictors of facility delivery and C-section were measured using multivariable analysis. As both of the variables were dichotomous, we used binary logistic regression models and the results expressed using odds ratios (OR) with 95%CIs. Each of the outcome variables were analyzed separately for the pooled, urban and rural participants. Model fit statistics were run after the regression analyses using the variance inflation factor (VIF) command. No multi-collinearity was detected as VIF values were below 10 for all the models. All tests were two-tailed and were considered significant at alpha value of 5%.

## Results

### Sample description

Table 1 shows that a greater proportion of the participants were aged 25–29 years (25.4%), rural residents (63.6%), had no education (61.6%), had no employment (41.39%), from households with poorer wealth quintile (24.7%), had access to electronic media (87.8%), didn’t hear about family planning on internet (98.8%), followers of Islam (98%), of Mandinka/Jahanka ethnicity (31.8%), had 1–5 children (76.2%), from male-headed households (82.%), and wanted the last child (84.7%).

**Table 1:** Sample characteristics

	*N* = 5351	%
**Age**		
15–19	370	6.91
20–24	1176	21.98
25–29	1359	25.40
30–34	1129	21.10
35–39	786	14.69
40–44	391	7.31
45–49	140	2.62
**Residency**		
Urban	1947	36.39
Rural	3,44	63.61
**Education**		
No Education	3,31	61.69
Primary	762	14.24
Secondary	1144	21.38
Higher	144	2.69
**Husband’s education**		
No Education	3459	67.30
Incomplete Primary	284	5.53
Incomplete Secondary	1087	21.15
Higher	310	6.03
**Occupation**		
Not Working	2205	41.39
Professional/Technical/Managerial	1276	23.95
Agricultural - Self Employed	1847	34.67
**Wealth index**		
Poorest	1264	23.62
Poorer	1325	24.76
Middle	1128	21.80
Richer	850	15.88
Richest	784	14.65
**Media access**		
No	645	12.15
Yes	4665	87.85
**Heard about FP on internet**		
No	5283	98.88
Yes	60	1.12
**Religion**		
Islam	5239	98.02
Other	106	1.98
**Ethnicity**		
Mandinka/Jahanka	1688	31.87
Wollof	747	14.10
Jola/Karoninka	397	7.49
Fula/Tukulur/Lorobo	1,39	26.24
Serahuleh	416	7.85
Other	659	12.44
**Parity**		
1 to 5	4082	76.28
> 5	1269	23.72
**Sex of household head**		
Male	4435	82.88
Female	916	17.12
**Wanted last child**		
Wanted Then	4525	84.75
Wanted No More	814	15.25
**ANC visits**		
< 4	1135	21.21
4 or more	4216	78.79

**Fig. 1:**
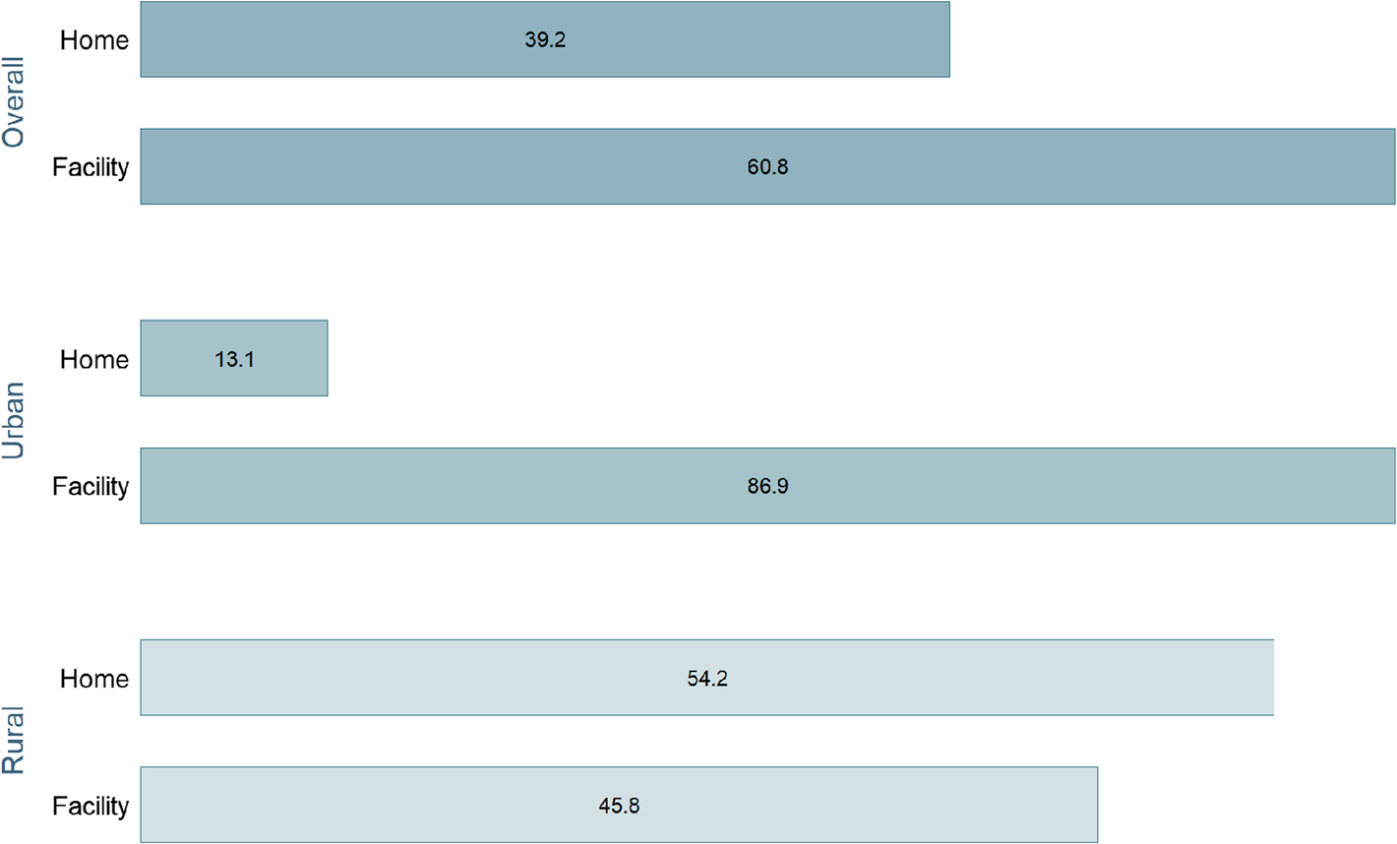
Prevalence of health facility delivery

Figure 1 shows that little over three-fifth (60.8%) of the participants had their last childbirth at a health facility and 39.2% at their home. There was a marked urban-rural difference in the prevalence of facility delivery (*p* = 0.014) with 86.9% of the urban women choosing health facility over home compared with 45.8% among the rural women.

**Fig. 2:**
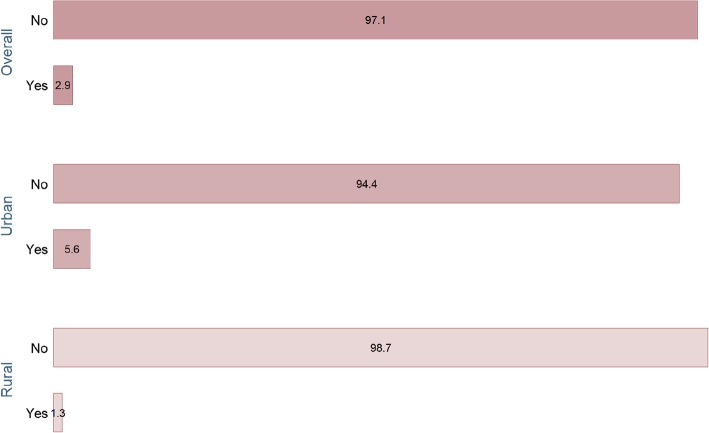
Prevalence of C-section delivery

As shown in Fig. 2, the overall prevalence of C-section delivery was quite low (2.9%). The figure also illustrates that urban women (5.6%) had a higer prevalence of choosing C-section compared with their rural (1.3%) counterparts.

The predictors of using facility delivery and C-section was presented in Tables 2 and 3 respectively. Table 2 indicates the odds of using facility delivery was lower among women with higher age groups. Compared with participants aged 15–19 years, those aged 20–24 [OR = 0.641, 95%CI = 0.478,0.859], 25–29 [OR = 0.496, 95%CI = 0.371, 0.663] and 30–34 [OR = 0.553, 95%CI = 0.409,0.749] years had lower odds of delivering at a health facility. This pattern was not significant for women aged above 39 years and those lived in urban areas. Rural residents had remarkably lower odds of using facility delivery [OR = 0.299, 95%CI = 0.238,0.375]. Having secondary [OR = 1.657, 95%CI = 1.337,2.053] and higher education [2.451, 95%CI = 1.166,5.150] showed positive association with the use of facility delivery services, especially among rural residents. Similar results were observed for husband’s education as well, with the odds being 1.485 [95%CI = 1.219,1.808] times higher for secondary and 1.628 times higher [95%CI = 1.136,2.333] for higher than secondary education. Compared with women without any employment, being employed in agriculture was negatively associated both in urban [OR = 0.577, 95%CI = 0.353,0.945] and in rural areas [OR = 0.477, 95%CI = 0.402, 0.567]. Regarding household wealth index, women from the richest wealth quintile had significantly higher [OR = 2.239, 95%CI = 1.525,3.289] odds of using facility delivery compared with those in the lowest quintile. Having access to electronic media showed a positive association [OR = 1.480, 95%CI = 1.201,1.824] among rural women only. Religion was not associated with using facility delivery services, while certain ethnic identities showed positive association. Having more than five childbirths showed a negative association [OR = 0.552, 95%CI = 0.356,0.854] using facility delivery. Households head’s sex and wantedness of child didn’t show in association. Women making adequate ANC visits had higher odds of using facility delivery services both in urban [OR = 1.619, 95%CI = 1.172,2.237] and rural [OR = 1.291, 95%CI = 1.075,1.550] areas.

**Table 2:** Predictors of using facility delivery services in Gambia

	Overall	Urban	Rural
**Age (15–19)**	1	1	1
20–24	0.641^**^ [0.478,0.859]	0.514 [0.219,1.210]	0.660^**^ [0.481,0.905]
25–29	0.496^***^ [0.371,0.663]	0.507 [0.218,1.182]	0.477^***^ [0.348,0.652]
30–34	0.553^***^ [0.409,0.749]	0.655 [0.275,1.557]	0.505^***^ [0.363,0.703]
35–39	0.689^*^ [0.491,0.965]	0.661 [0.267,1.633]	0.667^*^ [0.460,0.968]
40–44	0.999 [0.678,1.471]	1.112 [0.387,3.190]	0.917 [0.602,1.398]
45–49	1.109 [0.674,1.825]	0.939 [0.259,3.402]	1.090 [0.636,1.867]
**Residency (Urban)**	1		
Rural	0.299^***^ [0.238,0.375]		
**Education (No Education)**	1	1	1
Primary	1.119 [0.924,1.355]	1.199 [0.780,1.845]	1.089 [0.877,1.353]
Secondary	1.657^***^ [1.337,2.053]	1.267 [0.862,1.862]	1.830^***^ [1.412,2.373]
Higher	2.451^*^ [1.166,5.150]	1.364 [0.576,3.232]	6.380^*^ [1.420,28.67]
**Husband’s education (No Education)**	1	1	1
Incomplete Primary	1.031 [0.771,1.379]	1.110 [0.642,1.917]	0.949 [0.666,1.351]
Incomplete Secondary	1.485^***^ [1.219,1.808]	1.536^*^ [1.064,2.218]	1.449^**^ [1.145,1.835]
Higher	1.628^**^ [1.136,2.333]	1.644 [0.888,3.042]	1.612^*^ [1.033,2.514]
**Employment (Not Working)**	1	1	1
Professional/Technical/Managerial	0.981 [0.809,1.189]	0.903 [0.657,1.240]	1.022 [0.797,1.312]
Agricultural - Self Employed	0.478^***^ [0.408,0.560]	0.577^*^ [0.353,0.945]	0.477^***^ [0.402,0.567]
**Wealth quintile (Poorest)**	1	1	1
Poorer	0.948 [0.799,1.125]	1.177 [0.565,2.451]	0.946 [0.793,1.129]
Middle	0.789^*^ [0.655,0.951]	0.922 [0.512,1.659]	0.795^*^ [0.651,0.972]
Richer	1.090 [0.839,1.416]	1.596 [0.912,2.793]	0.812 [0.562,1.173]
Richest	2.239^***^ [1.525,3.289]	2.822^***^ [1.528,5.212]	6.782^*^ [1.439,31.96]
**Has media access (No)**	1	1	1
Yes	1.487^***^ [1.225,1.806]	1.135 [0.617,2.087]	1.480^***^ [1.201,1.824]
**Religion (Islam)**	1	1	1
Other	0.988 [0.521,1.872]	0.666 [0.241,1.843]	1.231 [0.567,2.673]
**Ethnicity (Mandinka/Jahanka)**	1	1	1
Wollof	1.051 [0.848,1.303]	2.477^**^ [1.366,4.489]	0.899 [0.704,1.148]
Jola/Karoninka	1.465^*^ [1.079,1.988]	1.204 [0.720,2.014]	1.650^**^ [1.136,2.397]
Fula/Tukulur/Lorobo	0.965 [0.814,1.145]	1.051 [0.704,1.567]	0.951 [0.785,1.151]
Serahuleh	0.944 [0.726,1.228]	1.073 [0.527,2.185]	0.906 [0.679,1.208]
Other	1.198 [0.929,1.544]	1.288 [0.848,1.956]	1.142 [0.822,1.585]
**Parity (1–5)**	1	1	1
> 5	0.854 [0.700,1.043]	0.552^**^ [0.356,0.854]	0.961 [0.767,1.204]
**Household head’s sex (Male)**	1	1	1
Female	1.174 [0.956,1.441]	1.032 [0.737,1.445]	1.207 [0.928,1.570]
**Child wantedness (Wanted Then)**	1	1	1
Wanted No More	0.838 [0.690,1.018]	0.903 [0.603,1.351]	0.819 [0.654,1.026]
**Antenatal Visits (< 4)**	1	1	1
4 or more	1.371^***^ [1.169,1.608]	1.619^**^ [1.172,2.237]	1.291^**^ [1.075,1.550]

Exponentiated coefficients; 95% confidence intervals in brackets

^*^p < 0.05, ^**^p < 0.01, ^***^p < 0.001

**Table 3:** Predictors of using caesarean section services in Gambia

	Overall	Urban	Rural
**Age (15–19)**	1	1	1
20–24	0.691 [0.284,1.682]	0.985 [0.267,3.633]	0.474 [0.130,1.737]
25–29	0.971 [0.418,2.255]	1.363 [0.389,4.775]	0.639 [0.191,2.141]
30–34	0.749 [0.310,1.808]	1.004 [0.278,3.627]	0.534 [0.141,2.024]
35–39	1.322 [0.531,3.294]	1.614 [0.435,5.985]	1.251 [0.304,5.155]
40–44	0.847 [0.274,2.621]	1.236 [0.270,5.661]	0.532 [0.0771,3.679]
45–49	3.341^*^ [1.049,10.64]	4.380 [0.834,23.00]	2.931 [0.526,16.35]
**Residency (Urban)**	1	1	1
Rural	0.619 [0.325,1.177]		
**Education (No Education)**	1	1	1
Primary	1.459 [0.874,2.435]	1.130 [0.586,2.182]	2.016 [0.883,4.599]
Secondary	1.284 [0.794,2.078]	0.981 [0.568,1.695]	2.609 [0.984,6.914]
Higher	2.122^*^ [1.002,4.493]	1.449 [0.640,3.283]	13.22^*^ [1.742,100.3]
**Husband’s education (No Education)**	1	1	1
Incomplete Primary	1.755 [0.928,3.319]	1.953 [0.913,4.180]	1.336 [0.381,4.679]
Incomplete Secondary	1.013 [0.644,1.595]	1.142 [0.675,1.930]	0.568 [0.200,1.614]
Higher	1.006 [0.514,1.969]	1.132 [0.535,2.396]	0.372 [0.0596,2.322]
**Employment (Not Working)**	1	1	1
Professional/Technical/Managerial	1.608^*^ [1.078,2.398]	1.245 [0.797,1.944]	3.839^**^ [1.576,9.350]
Agricultural - Self Employed	0.885 [0.501,1.561]	1.129 [0.445,2.867]	1.182 [0.504,2.775]
**Wealth quintile (Poorest)**	1	1	1
Poorer	0.734 [0.353,1.525]	0.937 [0.126,6.955]	0.666 [0.297,1.490]
Middle	1.401 [0.722,2.719]	1.547 [0.321,7.452]	1.294 [0.586,2.859]
Richer	1.276 [0.583,2.790]	1.632 [0.368,7.237]	1.202 [0.260,5.560]
Richest	3.014^**^ [1.373,6.615]	4.166 [0.950,18.27]	2.147^**^ [1.366,4.489]
**Has media access (No)**	1	1	1
Yes	0.708 [0.370,1.352]	0.392 [0.153,1.007]	0.879 [0.346,2.232]
**Religion (Islam)**	1	1	1
Other	0.991 [0.320,3.065]	1.196 [0.373,3.834]	0.965 [0.814,1.145]
**Ethnicity (Mandinka/Jahanka)**	1	1	1
Wollof	0.933 [0.524,1.662]	1.079 [0.539,2.158]	0.813 [0.271,2.442]
Jola/Karoninka	0.989 [0.511,1.912]	1.081 [0.498,2.347]	0.883 [0.241,3.236]
Fula/Tukulur/Lorobo	1.195 [0.738,1.933]	1.595 [0.865,2.943]	0.803 [0.360,1.790]
Serahuleh	1.056 [0.490,2.277]	1.071 [0.385,2.981]	1.105 [0.339,3.604]
Other	1.037 [0.602,1.789]	1.218 [0.659,2.249]	0.615 [0.133,2.840]
**Parity (1–5)**	1	1	1
> 5	0.853 [0.490,1.486]	0.796 [0.388,1.636]	0.844 [0.320,2.227]
**Household head’s sex (Male)**	1	1	1
Female	1.468 [0.992,2.172]	1.508 [0.976,2.328]	1.188 [0.438,3.220]
**Child wantedness (Wanted Then)**	1	1	1
Wanted No More	1.269 [0.808,1.993]	1.034 [0.586,1.827]	1.803 [0.840,3.870]
**Antenatal Visits (< 4)**	1	1	1
4 or more	1.443 [0.874,2.380]	1.753 [0.913,3.367]	1.023 [0.464,2.258]

Exponentiated coefficients; 95% confidence intervals in brackets

^*^p < 0.05, ^**^p < 0.01, ^***^p < 0.001

Regarding the use of C-section, age and residency didn’t show any statistically important difference, while having higher education [OR = 2.122, 95%CI = 1.002,4.493] showed positive association among rural residents. Women in the rural areas with a professional/managerial job had 3.839 times [OR = 1.576,9.350] higher odds of using C-section. Being in the richest wealth quintile also showed a positive association [OR = 3.014, 95%CI = 1.373,6.615], especially among rural [OR = 2.147, 95%CI = 1.366,4.489] women. Rest of the sociodemographic (e.g. ethnicity, child wantedness) and healthcare (ANC use) factors did not show any significant association.

## Discussion

Even though The Republic of The Gambia has made it a national priority to promote reproductive health the country still has one of the highest maternal mortality rates (597 deaths per 100,000 live births in 2017) in Sub-Saharan Africa. That being said, since the launch of the global Safe Motherhood Initiative in 1987, MMR has decreased mainly because the government has implemented a number of interventions (training and staffing of healthcare facilities, free healthcare services to all pregnant women, improvements in preventive and clinical services, etc.) to combat the dual scourge of neonatal and morbidity and mortality.

The present study is concerned about the pattern of using professional childbirth services, namely health and C-section. The results show that about two-fifth of the women in Gambia are deprived from facility delivery services. After taking into consideration the geographical factors, the situation looks even more challenging, with the prevalence being 45.8% among rural residents compared with 86.9% among the urbans. This striking disparity echoes the growing urban-rural inequality in health status and healthcare utilization across the continent. Women in the urban areas generally enjoy higher access to resources and better quality of services [14, 17]. Similar to facility delivery services, the use of C-section was also notably lower among rural residents (1.3% Vs 5.6%).

The beneficial aspects of C-sections have been highlighted in previous studies; however, it was reported the benefits generally plateau beyond the range of 19%. Results indicate that national prevalence of using C-sections in Gambia is far below the optimum range, and the African average as well. A global analysis on the trend in C-section between 1990 and 2014 reported that South America and the Caribbean region had the highest C-Section rates (40.5%) followed by Northern America (32.3%), Oceania (31.1%), Europe (25%), Asia (19.2%) with the African region having lowest rate (7.3%) of all [18]. The national and regional disparities, especially in contrast with Gambia and Africa provokes various theories relating the degree of socioeconomic development and healthcare infrastructure with the adoption of C-section. While a low prevalence of using C-sections can look positive from certain aspects, more data are necessary to understand whether the proportion of C-section in Gambia is in line with the actual need since the prevalence birth complications is expected to be comparative higher in underdeveloped countries.

In the multivariable analysis, significant sociodemographic differences were observed in the likelihood of the utilization of facility delivery services. Interestingly, higher age was associated negatively with facility delivery use, although increasing age is a good predictor of health literacy, education, decision-making power which are regarded as enabling factors of healthcare use [14, 19]. As expected from the univariate results, the odds of using facility delivery was lower in the rural areas compared with urban. The urban-rural difference in maternal healthcare use is a matter of serious public health concern in the countries with high maternal and child mortality rates. Women in the rural areas also have lower chances of recovery and survival from obstetric complications due to lower capacity and infrastructure. It is recommended that healthcare resource allocation is designed accordingly and puts stronger emphasis on rural and remote areas. We also observed that women with relatively higher education and wealth status had higher odds of using both facility delivery and C-section. This is understandable given the empowering quality of education and income generation, which in turn predict financial well-being and the ability to access healthcare services. Moreover, education can also influence women’s healthcare seeking behaviour through promoting health awareness and self-efficacy [20].

The use of C-section is comparatively less common in low-income settings, especially in countries like Gambia where maternal healthcare centers, especially those in the rural areas may not be equipped to the provide the service [21–23]. The reason behind this that C-section involves more sophisticated medical procedures and tools which can be made available through comprehensive MCH units only [24, 25]. As such, the availability of C-section services can vary substantially with lower accessibility in the rural and remote areas. Women living in rural settings and in marginalized conditions are less likely to afford C-section services, even when vaginal delivery is not recommendable in complicated cases [18, 26]. Therefore, special measures are necessary to ensure that the service is accessible to women based on need, regardless of geographic remoteness and women’s capacity to pay.

The results further indicate that women in agrarian communities are more deprived of facility delivery services compared even with non-working women. The financial and livelihood hardship endured by women employed in agriculture is well-known. It is therefore recommended that maternal healthcare promotion programs pay special attention to farming communities. Several other factors were found to be significantly associated with facility delivery service utilization that merit special attention including media access, higher parity and adequate use of ANC services. Mass media communication plays a key role in promoting health knowledge and awareness among general population, and can help promote the use of maternity services in low-income settings. The positive association between media access and facility delivery service utilization in the present study suggests that investing on media exposure and quality healthcare communication can improve the uptake of the services. Media exposure can equally raise awareness regarding family planning services and help women gaining control over their reproductive preferences. As shown by the results, women with higher parity are less likely to use facility delivery services. Lastly, using ANC also showed significantly positive association with facility delivery service utilization. Women who visit healthcare professionals during pregnancy are generally more likely to gain knowledge about the dangers of unattended childbirth, consequences of complicated pregnancy, and understand the importance of choosing skilled birth services. In light of this finding, it is recommended to improve the coverage of ANC services which may increase the use of professional childbirth services.

This study is the first to report the prevalence of using facility delivery and C-section use in nationally-representative sample in Gambia. Important sociodemographic disparities were observed in the odds of using the services, among which the most notable were women’s education, ethnic background, wealth status of the household, parity, and family planning communication through mass media. These findings of the present study fill an important gap in the literature. Main strengths were the use of nationally representative data from four rounds of survey that allows making a generalizable conclusion about the prevalence and associations. Nonetheless, this study has several limitations to report. First of all, the data were cross-sectional and hence no causality can be inferred from the associations. The data were secondary and authors have to influence over the selection and measurement of the variables. As the data were self-reported, the chances of recall and reporting bias cannot be ignored. The factors that influence healthcare service utilization are diverse and multifaceted, but the choice of the explanatory factors was limited as the survey was not conducted by the authors. Factors such as geographical distance, transportation facilities, quality of services in local healthcare settings, availability of female care provider have been found to be important in primary studies, that we were not able to adjust for in the current analysis. The use of C-sections is also a complex outcome that can be driven by various personal and medical factors. However, no such data were collected in this survey. For large-scale surveys like the GDHS, collecting in-depth data may not always be feasible due to time and resource constraints. Future studies should aim to address these limitations by considering more sociocultural and environmental variables. Lastly, due to the nature of the survey and analyses, causality cannot be inferred for the relationship between the outcome and explanatory factors.

## Conclusion

Finding of the present study reflect sub-optimal use of professional childbirth services in Gambia. The factors behind this scenario can be multifactorial and require in-depth investigations. As implied by the findings, the differences might be rooted in geographical, educational, wealth inequality, parity and low use of ANC services. The role of socioeconomic and demographic factors in accessing childbirth services are well-supported by the present study. Based on these insights, it is recommended that health policy makers establish special intervention programs focusing on socially disadvantaged communities with the aim of reducing maternal health related inequalities, which can also pay off for the actions towards attaining Sustainable Development Goals (SDGs).

## Data Availability

Data for this study were sourced from Demographic and Health surveys (DHS) and available here: http://dhsprogram.com/data/available-datasets.cfm.

## Abbreviations

DHS: Demographic and Health Surveys
MCM: Maternal and child mortality
MDGs: Millennium Development Goals
SBAs: Skilled birth attendants
SDGs: Sustainable Development Goals
WHO: World Health Organization
